# Polyhydroxycurcuminoids but not curcumin upregulate neprilysin and can be applied to the prevention of Alzheimer’s disease

**DOI:** 10.1038/srep29760

**Published:** 2016-07-13

**Authors:** Po-Ting Chen, Zih-ten Chen, Wen-Chi Hou, Lung-Chih Yu, Rita P.-Y. Chen

**Affiliations:** 1 Institute of Biochemical Sciences, National Taiwan University, No. 1, Sec. 4, Roosevelt Rd., Taipei 106, Taiwan; 2 Institute of Biological Chemistry, Academia Sinica, No. 128, Sec. 2, Academia Rd., Nankang, Taipei 115, Taiwan; 3 Graduate Institute of Pharmacognosy, Taipei Medical University, No. 250, Wuxing St., Taipei 110, Taiwan

## Abstract

Neprilysin (NEP) is the most important Aβ-degrading enzyme. Its expression level decreases with age and inversely correlated with amyloid accumulation, suggesting its correlation with the late-onset of Alzheimer’s disease. Recently, many reports showed that upregulating NEP level is a promising strategy in the prevention and therapy of Alzheimer’s disease. Here, we used a sensitive fluorescence-based Aβ digestion assay to screen 25 curcumin analogs for their ability to upregulate NEP activity. To our surprise, four compounds, dihydroxylated curcumin, monohydroxylated demethoxycurcumin, and mono- and di-hydroxylated bisdemethoxycurcumin, increased NEP activity, while curcumin did not. The ability of these polyhydroxycurcuminoids to upregulate NEP was further confirmed by mRNA and protein expression levels in the cell and mouse models. Finally, feeding monohydroxylated demethoxycurcumin (also named demethylcurcumin) or dihydroxylated bisdemethoxycurcumin (also named bisdemethylcurcumin) to APP_swe_/PS_1_dE_9_ double transgenic mice upregulated NEP levels in the brain and reduced Aβ accumulation in the hippocampus and cortex. These polyhydroxycurcuminoids offer hope in the prevention of Alzheimer’s disease.

Age-related Alzheimer’s disease (AD), the most common form of dementia, is characterized by the presence of intracellular neurofibrillary tangles and extracellular senile plaques. The senile plaques are mainly composed of amyloid-β peptides (Aβ), which are 39–43 amino acid peptides. According to the amyloid cascade hypothesis, the excessive accumulation and abnormal aggregation of Aβ is linked to the onset of the neurodegenerative process. Recent success in the clinical trial of passive anti-Aβ immunotherapy such as Aducanumab on reducing Aβ accumulation and slowing cognitive decline in prodromal and mild AD patients suggested that Aβ clearance is a promising strategy in AD prevention and therapy^1^,^2^.

Aβ levels are a dynamic equilibrium between production and clearance. Under normal physiological condition, Aβ can be degraded by several endogenous endopeptidases including neprilysin (NEP), plasmin, insulin-degrading enzyme (IDE), endothelin-converting enzyme, angiotensin-converting enzyme (ACE), and several matrix metalloproteases^3^. NEP has been singled out as the most promising target because (1) its protein levels are lower in AD brains than normal brains^4^ and are inversely correlated with age^5^; (2) NEP mRNA levels are lower in high plaque density regions of human AD brains than in other regions or the corresponding regions of normal brains^6^; (3) NEP activity and protein levels in the hippocampus decline with age in mice^7^; (4) NEP protein levels are higher in the cerebellum of mice than in the cortex and hippocampus, regions of major Aβ plaque accumulation^7^,^8^; and (5) Aβ levels are twice as high in NEP knock out mice^3^,^9^. It has been reported that peripheral overexpression of NEP in muscle^10^ or leukocytes^11^, direct injection of NEP protein into the brain^12^, or overexpression NEP in the brain of AD transgenic mice^13^,^14^,^15^ reduced amyloid load in the mouse brain and improved cognitive ability. These data strongly link NEP activity with amyloid accumulation in AD pathogenesis. Since NEP expression declines with age^5^, restoring NEP activity to its normal level or maintaining its normal level would be beneficial in restoring or maintaining homeostasis of Aβ production and degradation and could serve as a feasible approach for AD prevention.

Certain medicinal plants are thought to be effective in improving brain function, and secondary metabolites in plants have been extensively examined as possible AD therapeutic reagents. Ayoub and Melzig found that apigenin, luteolin, and curcumin increased both ACE and NEP activity and rolipram increased only NEP activity in human SK-N-SH neuroblastoma cells^16^. Eisele *et al*. reported that Gleevec (a tyrosine kinase inhibitor) treatment increased NEP protein and mRNA levels in human APP transfected H4 human neuroglioma cells^17^. Klein *et al*. reported that kynurenic acid (a tryptophan catabolite) induced NEP protein and mRNA levels and also NEP activity in human SH-SY5Y neuroblastoma cells^18^. AD incidence surveys have reported that the incidence of AD in an Indian population was almost 5 times lower than that in a US population^19^,^20^. Turmeric is widely used as a food additive (curry spice) and as a traditional medicine in Asia, particular in India. Since diet is one of the causative factors for AD, these surveys stimulated many studies exploring the effect of curcumin or curcuminoids (the major effective components in turmeric) and their possible application in AD therapy^21^. Curcuminoids are a mixture of bisdemethoxycurcumin (3–5%), demethoxycurcumin (15–20%), and curcumin (75–80%)^22^. Liao *et al*.^23^ showed that curcuminoids promote neurite outgrowth in PC12 cells. Lim *et al*.^24^ fed curcumin to an AD transgenic mouse model (Tg2576) for 6 months and found that low-dose curcumin treatment (160 ppm; ~32 mg/kg/day) reduced Aβ content and plaque burden in the brain, while high-dose treatment (5000 ppm; ~990 mg/kg/day) had no effect. Wang *et al*.^25^ showed that feeding a high dose of curcumin (200 mg/kg/day) to an AD double transgenic mouse model (APP_swe_/PS_1_dE_9_) for 3 months reduced Aβ levels and increased NEP and IDE expression in the hippocampus and improved spatial learning and memory ability. Ahmed *et al*.^26^ reported that intraperitoneally injected curcuminoids enhanced memory in amyloid-infused rats; interestingly, in their study, bisdemethoxycurcumin and demethoxycurcumin were more effective than curcumin. It has been known that curcumin has very poor solubility and bioavailability and demethoxylation could improve solubility^27^. Curcumin analogs with better bioavailability might have better efficacy. We hypothesize that turmeric might have beneficial effect on maintaining the balance between anabolism and catabolism of Aβ by upregulating NEP and other curcumin analogs in turmeric might be more beneficial than curcumin.

## Results

### Screen of curcumin analogs on upregulation of NEP by activity assay

**Figure 1:**
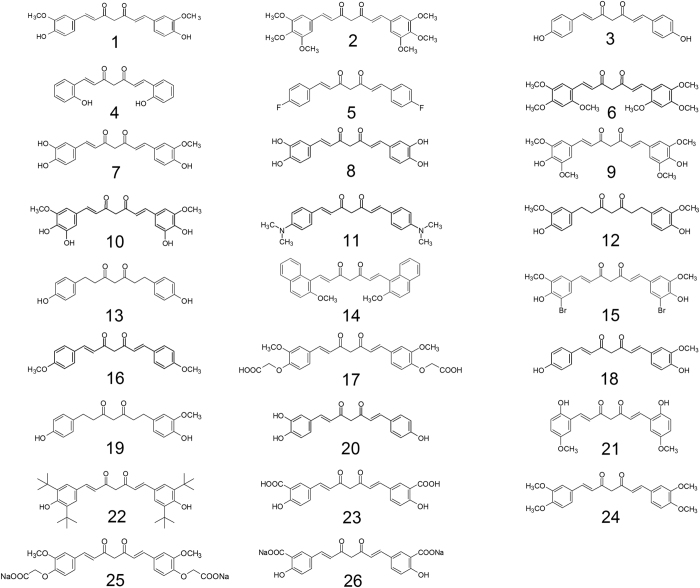
Structures of curcumin (compound 1) and 25 curcumin analogs (compound 2-26). 1, 1,7-Bis(4-hydroxy-3-methoxyphenyl)-1,6-heptadiene-3,5-dione); 2, 1,7-Bis(3,4,5-trimethoxyphenyl)-1,6-heptadiene-3,5-dione; 3, 1,7-Bis(4-hydroxyphenyl)-1,6-heptadiene-3,5-dione; 4, 1,7-Bis(2-hydroxyphenyl)-1,6-heptadiene-3,5-dione; 5, 1,7-Bis(4-fluorophenyl)-1,6-heptadiene-3,5-dione; 6, 1,7-Bis(2,4,5-trimethoxyphenyl)-1,6-heptadiene-3,5-dione; 7, 1-(3,4-Dihydroxyphenyl)-7-(3-methoxy-4-hydroxyphenyl)-1,6-heptadiene-3,5-dione; 8, 1,7-Bis(3,4-dihydroxyphenyl)-1,6-heptadiene-3,5-dione; 9, 1,7-Bis(4-hydroxy-3,5-dimethoxyphenyl)-1,6-heptadiene-3,5-dione; 10, 1,7-Bis(3,4-dihydroxy-5-methoxyphenyl)-1,6-heptadiene-3,5-dione; 11, 1,7-Bis(4-N,N-dimethylaminophenyl)-1,6-heptadiene-3,5-dione; 12, 1,7-Bis(4-hydroxy-3-methoxyphenyl)heptan-3,5-dione; 13, 1,7-Bis(4-hydroxyphenyl)heptan-3,5-dione; 14, 1,7-Bis(2-methoxy-1-naphthyl)-1,6-heptadiene-3,5-dione; 15, 1,7-Bis(3-bromo-4-hydroxy-5-methoxyphenyl)-1,6-heptadiene-3,5-dione; 16, 1,7-Bis(4-methoxyphenyl)-1,6-heptadiene-3,5-dione; 17, 1,7-Bis(3-methoxy-4-carboxymethoxyphenyl)-1,6-heptadiene-3,5-dione; 18, 1-(4-Hydroxyphenyl)-7-(4-hydroxy-3-methoxyphenyl)-1,6-heptadiene-3,5-dione; 19, 1-(4-Hydroxyphenyl)-7-(4-hydroxy-3-methoxyphenyl)-heptan-3,5-dione; 20, 1-(3,4-Dihydroxyphenyl)-7-(4-hydroxyphenyl)-1,6-heptadiene-3,5-dione; 21, 1,7-Bis(2-hydroxy-5-methoxyphenyl)-1,6-heptadiene-3,5-dione; 22, 1,7-Bis(3,5-di-*tert*-butyl-4-hydroxyphenyl)-1,6-heptadiene-3,5-dione; 23, 1,7-bis(3-carboxy-4-hydroxyphenyl)-1,6-heptadiene-3,5-dione; 24, 1,7-Bis(3,4-dimethoxyphenyl)-1,6-heptadiene-3,5-dione; 25, 1,7-Bis(4-carboxymethoxy-3-methoxyphenyl)-1,6-heptadiene-3,5-dione disodium salt; 26, 1,7-bis(3-carboxy-4-hydroxyphenyl)-1,6-heptadiene-3,5-dione disodium salt.

**Figure 2:**
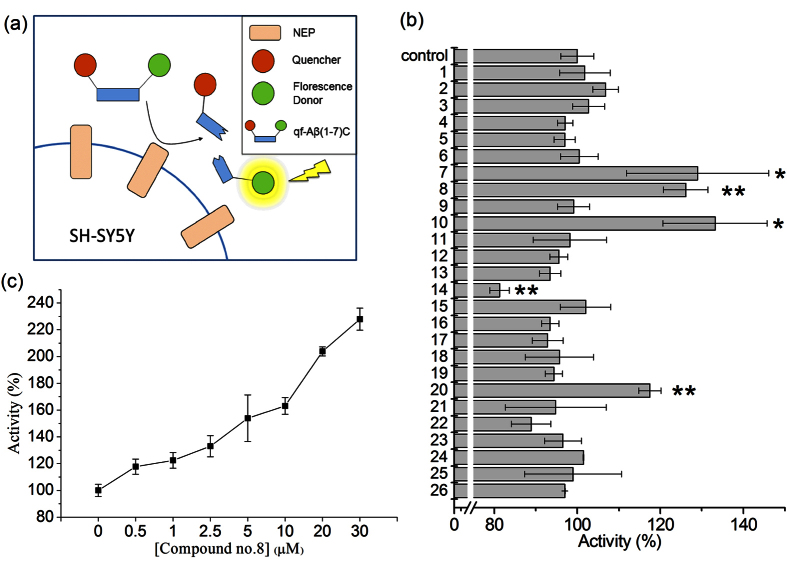
Screen compounds with the ability to increase Aβ-degradation. (**a**) Aβ-degrading activity assay using qf-Aβ(1-7)C as substrate. (**b**) Results for SH-SY5Y cells incubated for 24 h with 0.5% DMSO (control) or the compound listed on the y axis (5 μM). (**c**) Aβ-degrading activity assay using different concentrations of compound 8. In (**b,c**), the data are presented as the mean ± SD for three independent samples; in (**b**) *p < 0.05; **p < 0.01 compared to the control by one-way ANOVA.

We first compared the ability of curcumin and 25 curcumin analogs to enhance NEP activity using human SH-SY5Y cells; the structures of these compounds are shown in Fig. 1. In this activity assay, we measured the fluorescence increase caused by cleavage of a quenched fluorogenic peptide, qf-Aβ(1-7)C, which can be cleaved by NEP and IDE, but not by other Aβ-degrading enzymes (Fig. 2a)^28^. Since NEP is an ectoenzyme, after the cells were treated with compounds, the compounds were removed by replacing the cell medium with the assay buffer containing qf-Aβ(1-7)C which can be degraded by NEP on cell surface. The fluorescence intensity in the assay buffer was measured. The results showed that compounds 7 (monohydroxylated demethoxycurcumin, also named demethylcurcumin), 8 (dihydroxylated bisdemethoxycurcumin, also named bisdemethylcurcumin), 10 (dihydroxylated curcumin), and 20 (monohydroxylated bisdemethoxycurcumin, also named demethylmonodemethoxycurcumin) enhanced fluorescence intensity whereas curcumin did not (Fig. 2b) and that the fluorescence increase, using compound 8 as an example, was dose-dependent (Fig. 2c). Comparison of the results for compound 1(curcumin; with 2 methoxy groups), compound 3 (bisdemethoxycurcumin; without methoxy group), compound 9 (with 4 methoxy groups), and compound 18 (demethoxycurcumin; with one methoxy group) showed that the number of methoxy groups is not relevant to their efficacy on upregulating NEP activity in SH-SY5Y cells. Comparing the structures of four effective compounds examined (compound 7, 8, 10, 20) with the others, we found that the effective compounds have at least three hydroxyl groups (three for compounds 7 and 20 and four for compounds 8 and 10) on the 1,7-bisphenyl-1,6-heptadiene-3,5-dione scaffold. We therefore propose that the number of hydroxyl groups on curcumin analogs is critical in efficacy of NEP upregulation.

### Compounds 7, 8, 10, 20 upregulate NEP rather than other Aβ-degrading enzymes

**Figure 3:**
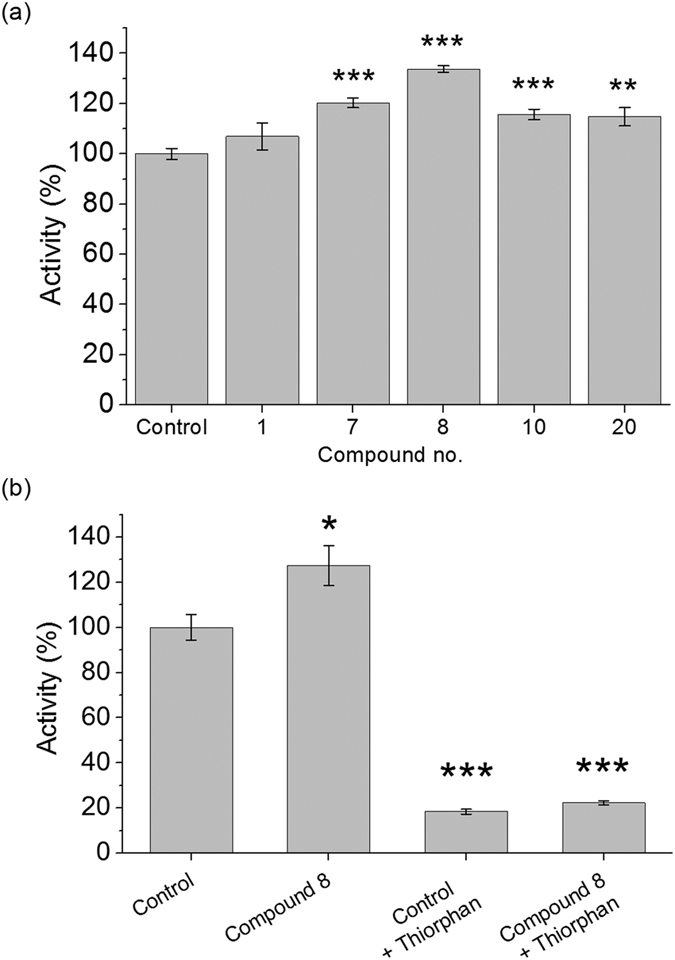
Examination of the increased Aβ-degrading activity coming from the action of NEP. (**a**) Aβ-degrading activity assay using qf-Aβ(12-16)AAC as substrate. SH-SY5Y cells were incubated 12 h with 0.5% DMSO (control) or the indicated compounds (5 μM) before assay. (**b**) Thiorphan inhibition assay. SH-SY5Y cells were incubated for 12 h with 0.5% DMSO or compound 8 (5 μM), then with or without 50 μM thiorphan for 30 min before Aβ-degrading activity was measured. The data are the mean ± SD for three independent samples. *p < 0.05; **p < 0.01; ***p < 0.001 compared to the control by Student’s t-test.

**Figure 4:**
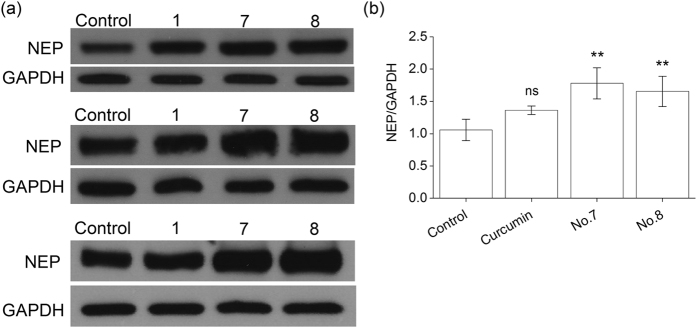
Western blot analysis and quantitative densitometry for NEP levels in RA-differentiated SH-SY5Y cells. The cells were incubated with 0.5% DMSO (control) or 5 μM curcumin (no. 1) or compound 7 or 8 for 24 h, then NEP protein in the cell membrane fraction was measured by western blotting. (**a**) Western blotting results of three independent experiments. GAPDH was used as the loading control. (**b**) The quantification of NEP protein levels by Image J. The NEP intensities were normalized to the GAPDH intensities for three independent experiments. Data are presented as the mean ± SD; ns, not significant; **p < 0.01 compared to the DMSO-treated control group by Student’s t-test.

Since qf-Aβ(1-7)C can be cleaved by both NEP and IDE^28^, whether the enhanced fluorescence intensity indeed came from the reaction of NEP or IDE was examined by using another substrate-specific peptide substrate. The peptide, qf-Aβ(12-16)AAC, which can be cleaved by ACE and NEP, but not other Aβ-degrading enzymes^29^, was used to test the effects of compounds 1, 7, 8, 10, and 20. As shown in Fig. 3a, compounds 7, 8, 10, and 20 caused increased activity, suggesting that these four compounds activate NEP, and not IDE. This conclusion was confirmed in the case of compound 8 using thiorphan, an NEP-specific inhibitor (Fig. 3b). After incubating SH-SY5Y cells with or without compound 8 for 12 h, the cell medium was replaced with 400 μL of assay buffer alone or containing 50 μM thiorphan for 30 min prior to addition of qf-Aβ(12-16)AAC, and thiorphan was found to decrease substrate cleavage activity to about 20% of the untreated control, both in the absence or presence of compound 8, demonstrating that most of the increased activity induced by compound 8 was due to an effect on NEP. Moreover, we examined the NEP protein level in the membrane fraction of the differentiated SH-SY5Y cells without or with treatment of curcumin, compound 7, or compound 8 by western blotting. Compounds 7 and 8 significantly increased NEP protein (Fig. 4).

### Compounds 7 & 8 increase NEP mRNA levels in the mouse brain

**Figure 5:**
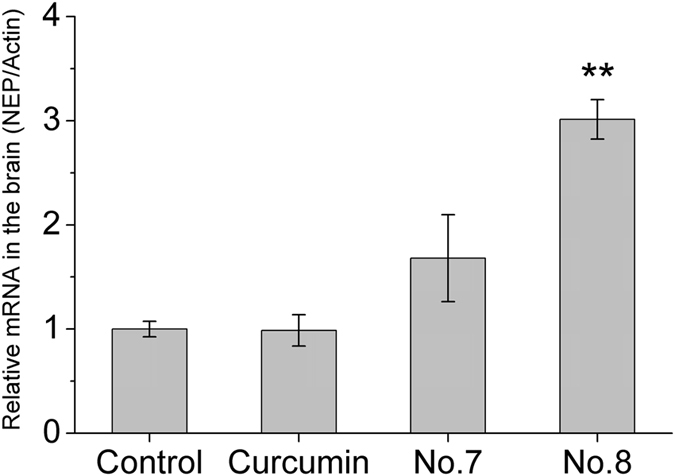
NEP mRNA levels in the brains of mice fed vehicle (control) or the indicated compound for 7 days (10 mg/kg BW per day) by gavage (n = 5). β-actin was used as the reference gene. The results are the mean ± SEM; **p < 0.01 compared to the vehicle-fed group by Student’s t-test.

To examine whether compound 7 & 8 can pass blood-brain-barrier and function in the brain, we fed curcumin or compound 7 or 8 to normal B6C3 mice (10 mg/kg BW) every day for 7 days. The total NEP mRNA level in the brain was quantitated by real-time PCR. The results showed that feeding compound 7 or 8 increased NEP mRNA level in the mouse brain, whereas feeding with curcumin did not yield the same effect (Fig. 5).

### Compound 7 upregulates NEP and decreases Aβ accumulation in the hippocampus and cortex of the AD double transgenic mice APP_swe_/PS_1_dE_9_

**Figure 6:**
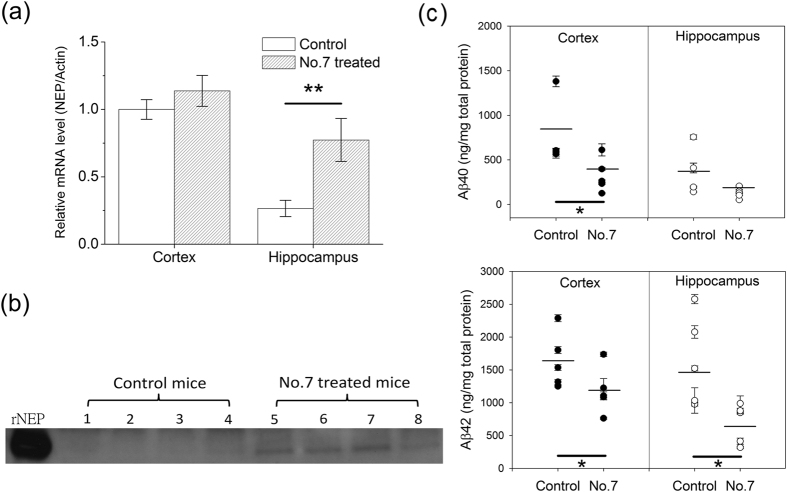
Effect of compound 7 on NEP mRNA and protein levels and Aβ clearance *in vivo*. APP_swe_/PS_1_dE_9_ mice were fed vehicle (control) or compound 7 (10 mg/kg BW) six times per week for 6.5 months by gavage. (**a**) NEP mRNA levels in the cortex and hippocampus using β-actin as the reference gene, with the value for the cortex of the vehicle-fed control group set as 1. The results are presented as the mean ± SEM (n = 5). (**b**) Neprilysin western blot. Protein samples of 12 μg extracted from the hippocampus of eight APP_swe_/PS_1_dE_9_ mice fed with vehicle or compound 7 were resolved on an 8% Bis-Tris gel and immunoblotted with rat monoclonal anti-mouse NEP antibody. (**c**) ELISA results for formic acid-extracted (insoluble) Aβ40 (top panel) and Aβ42 (bottom panel) in the cortex and hippocampus; the data for each mouse are indicated and the mean is indicated by the line (n = 5). The heavy black line indicates the sets of data showing a significant difference; *p < 0.05; **p < 0.01 compared to the control by Student’s t-test.

Compound 7 is a minor component of turmeric, i.e. a natural compound^30^ and has been reported with a higher antioxidative effect than curcumin by inhibiting lipid peroxidation and protein oxidation in rat liver mitochondria^31^. It was therefore tested for efficacy in Aβ clearance in double transgenic APP_swe_/PS_1_dE_9_ mice. Mice aged 4.5–5.5 months were fed compound 7 (10 mg/kg BW) or vehicle by gavage six times a week at a much lower dose than that of curcumin used in previous studies^24^,^25^,^32^,^33^. After feeding for 6.5 months, the mice were sacrificed, and then NEP mRNA levels in the hippocampus and cortex were quantitated by real-time PCR (Fig. 6a). It is clear that NEP mRNA level in the hippocampus was lower than that in the cortex of the untreated mice. After treating with compound 7, a significant increase could be seen in the hippocampus with a slight increase in the cortex. The western blotting data in Fig. 6b also showed that the NEP protein amount in the cortex was higher in the mice fed with compound 7 than in the mice fed with vehicle. As a consequence, compound 7 feeding should be able to decrease Aβ accumulation in the brain. The Aβ amounts were analyzed by ELISA. The compound 7-treated mice had a significant decrease in formic acid-extracted insoluble Aβ40 (top panel) and Aβ42 (bottom panel) in the cortex and hippocampus (Fig. 6c).

### Compound 8 reduced Aβ plaque load in the brain of the AD double transgenic mice APP_swe_/PS_1_dE_9_

**Figure 7:**
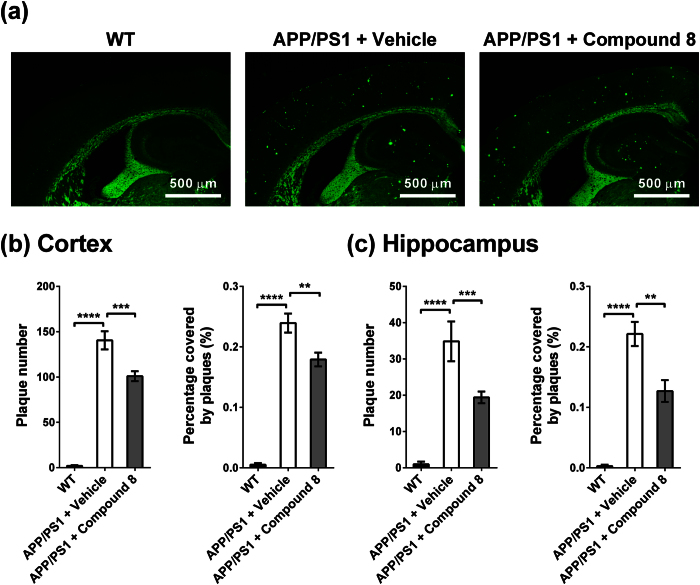
Effect of compound 8 on reducing amyloid plaque burden in the mouse brain. The APP_swe_/PS_1_dE_9_ mice were treated with vehicle or compound 8 for 8 months. Plaque burden of the 11-month-old WT littermate (n = 4), vehicle-treated (n = 3) and compound 8-treated transgenic mice (n = 6) were analyzed by ThS staining (4 slices per mouse). (**a**) Representative ThS-staining images. (**b,c**) Plaque number and percentage of plaque-covered area in the cortex (**b**) and hippocampus (**c**) were quantitated by ImageJ. Data was expressed in mean ± SEM (*p < 0.05, **p < 0.01, ***p < 0.001, ****p < 0.0001 by one-way ANOVA with Fisher’s LSD test).

Mice aged 3 months were fed compound 8 (10 mg/kg BW) or vehicle by gavage six times a week for 8 months. The amyloid plaque accumulation was then evaluated by ThS staining (Fig. 7). The amyloid plaque number in the compound 8-treated transgenic mice was reduced by 28% in the cortex and 44% in the hippocampus. The plaque-covered area was reduced by 25% in the cortex and 43% in the hippocampus. The data indicated that feeding compound 8 was effective in decreasing plaque burden in the brain, especially in the hippocampus. This observation aligns with our NEP mRNA quantification results in Fig. 5. Compound 8 can increase NEP mRNA level. It has been reported that the hippocampus has the lowest NEP expression compared with other regions in the brain of mouse and human^7^,^34^. NEP expression might be under well-regulated control, so its expression cannot increase much to degrade other neuropeptides. Therefore, the upregulation effect of compound 8 is more prominent in the region that has lower NEP expression, i.e. hippocampus, and the amyloid load reduction is greater in the hippocampus than cortex.

### Possible mechanism of polyhydroxycurcuminoids-induced NEP upregulation

**Figure 8:**
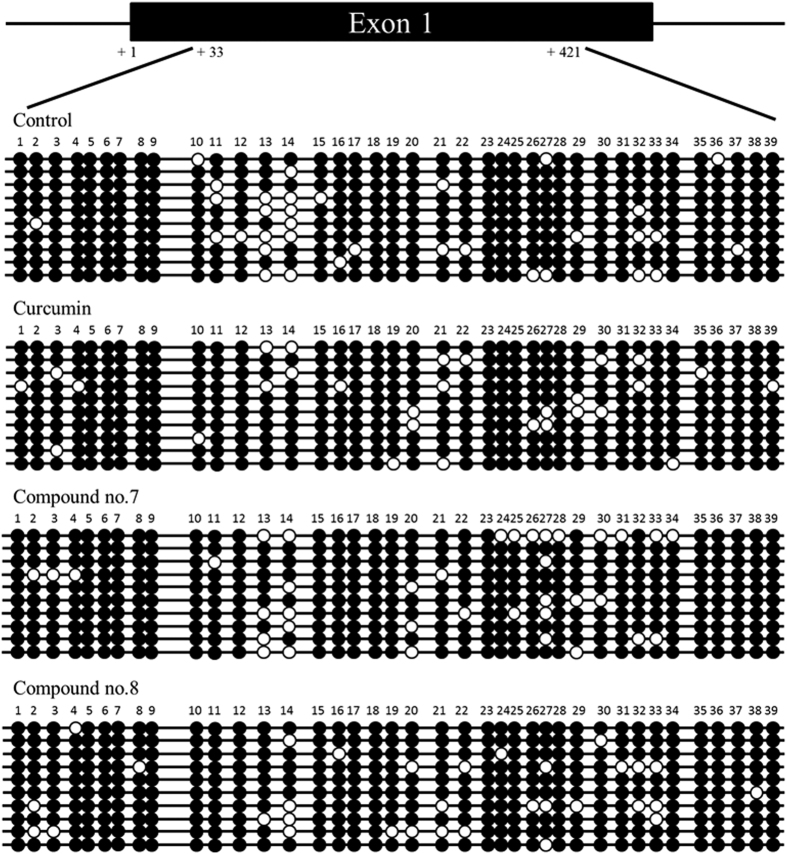
Effects of curcumin and compounds 7 and 8 on DNA methylation of NEP in the N2a cells. The genomic structure of the CpG island in the mouse NEP promoter region is drawn to scale above the bisulfite sequencing chromatogram. The CpG sites are indicated by number. Each row of circles indicates a single cloned allele. Black circle, methylated cytosine; white circle, unmethylated cytosine.

It has been reported that NEP transcription can be regulated by DNA methylation in a CpG island in its promoter region, and that hypermethylation in this CpG island is related to decreased NEP expression in various cancers^35^,^36^. Here, we examined whether polyhydroxycurcuminoids-induced NEP upregulation is achieved via altered NEP DNA methylation status. The N2a cells were treated with curcumin, compound 7, or compound 8, and the methylation of the NEP gene was analyzed by bisulfite genomic sequencing according to the literature^37^. In 10 clones, the percentages of total methylated CpG were 91.3 (control), 92.6 (curcumin), 90.5 (compound 7), and 91.8% (compound 8) (Fig. 8). The NEP gene was heavily methylated in all four groups. Compound 7 and 8 treated cells have slightly more sites (20 sites) with unmethylated cytosine than control (18 sites) and curcumin treated cells (19 sites). When comparing only the high CpG density region (site 23–34), the percentages of total methylated CpG were 92.5 (control), 91.7 (curcumin), 84.2 (compound 7), and 89.2% (compound 8). The differences were small, except for compound 7 (p = 0.069 compared with control by Fisher’s exact test). None of the compound-treated groups showed significant difference compared with control. We did not observe a significant change in CpG demethylation in the curcumin treated group, which was reported by Deng *et al*.^37^. Compounds 7- and 8- treated groups have less methylated CpG than curcumin-treated but the differences were not statistically significant (see supplementary information). Based on the data, we can not exclude the possibility that CpG demethylation is involved in the polyhydroxycurcuminoids-induced NEP upregulation, but other mechanisms, such as NEP oxidation^4^, NEP mRNA stability^38^, NEP membrane translocation^39^, might also contribute to the NEP upregulation.

## Discussion

Previously, somatostatin, rolipram, luteolin, apigenin, and curcumin was reported showing the ability of increasing NEP activity using activity assay but there was no supporting data showing increase in NEP mRNA or protein levels^16^,^40^. Using activity assay alone could not exclude the possibility the substrate degradation coming from the action of other proteases. Gleevec and kynurenic acid have been reported with the ability of increasing NEP protein and mRNA levels^17^,^18^ in the cell models. However, no *in vivo* data to support that they can upregulate NEP in the brain. In our study, we employed two different activity assays and one NEP inhibitor assay to corroborate that our screened compounds are effective on upregulating NEP but not on other proteases. The assay of mRNA levels in the mouse brain tissues proved that our screened compounds which were delivered orally can pass blood-brain-barrier to upregulate NEP in the brain. We noted that this upregulation was not dramatic but mild. We surmise that there might be a feedback inhibition mechanism to regulate NEP transcription to avoid big increase in NEP level. The significant difference in NEP mRNA level after our compound 7 treatment occurs in the hippocampus where the mRNA level is much lower than that in the cortex. It has been reported that NEP activity and protein levels in the hippocampus decline with age in mice^7^,^8^. Increasing NEP level in hippocampus should be very beneficial to rebuild the balance between Aβ production and degradation and avoid Aβ accumulation in the hippocampus where is important for memory.

In this study, curcumin was not found to affect NEP activity, mRNA levels, or protein levels. Other major components in turmeric such as demethoxycurcumin and bisdemethoxycurcumin could not increase NEP activity either. The screened effective curcumin analogs have three or more hydroxyl groups. These polyhydroxycurcuminoids, such as compounds 7 and 20, were found in curcuminoids extracted from rhizomes of *Curcuma longa*^30^,^41^. We propose that the most effective components in turmeric for AD prevention are the hydroxylated curcumin and (bis)demethoxycurcumin (i.e. polyhydroxycurcuminoids), but not the major components curcumin and (bis)demethoxycurcumin. Curcumin is well known with poor solubility and bioavailability. Because these polyhydroxycurcuminoids contain more hydrophilic hydroxyl groups, their solubility and bioavailability should be greater than those of curcumin^27^. Moreover, it has been reported that polyhydroxycurcuminoids have higher antioxidant activity than curcumin and other commercially available antioxidants, such as vitamin C and vitamin E^42^,^43^. We conclude that these polyhydroxycurcuminoids are better candidates than curcumin as a dietary supplement in the prevention and therapy of Alzheimer’s disease.

## Methods

### Screened compounds

Curcumin (no.1) and 25 curcumin analogs (no. 2–26) were purchased from Laila Impex Co. (Vijayawada, India) with purity >99%. The names and structures of these compounds are shown in Fig. 1.

### Cell culture

Human neuroblastoma SH-SY5Y cells were purchased from the American Type Culture Collection (ATCC, USA) and cultured in Dulbecco’s modified Eagle’s medium: Nutrient Mixture F-12 (DMEM/F-12, Life technologies, USA) supplemented with 10% fetal bovine serum (FBS; Biological industries, USA) in 5% CO_2_ at 37 °C.

### Aβ-degrading activity assay using qf-Aβ(1-7)C as substrate

Test compounds 1–24 were dissolved as stock solutions of 1–10 mM in DMSO and compounds 25 and 26 were dissolved as a 1 mM stock solution in distilled water and diluted in medium for use; the vehicle control, DMSO was diluted to the same extent (0.2–0.5%). qf-Aβ(1-7)C (sequence DAEFRHDC with Alexa-350 (Alexa Fluor® 350 C_5_-maleimide, Invitrogen) conjugated to the sidechain of Cys and amine-reactive Dabcyl (4-(4′-N,N-dimethylaminophenyl)azobenzoic acid, succinimidyl ester, Invitrogen) as a quencher linked to the N-terminus of the peptide) was synthesized^28^ and was dissolved in DMSO as a 2 mM stock solution. For screening, 96-well culture plates were used to screen the 26 compounds. SH-SY5Y cells were grown to confluence in 100 mm culture dishes, then were seeded in 96-well plates (200 μL; cell density 5 × 10^5^ cells/mL). After plating for 1 day, the medium was replaced with fresh DMEM medium supplemented with 10% FBS with or without the indicated compound (final concentration 5 μM), then the cells were incubated for 24 h, after which the medium was replaced with 200 μL assay buffer (PBS containing 5.5 mM D-glucose, 0.3 mM sodium pyruvate, 25 mM sodium bicarbonate, and 1.5 μM zinc sulfate) containing 2 μM qf-Aβ(1-7)C and the cells incubated for 1.5 h. To measure Aβ-degrading activity, 150 μL of the assay buffer was taken for fluorescence measurement on a SpectraMax Gemini EM (Molecular Devices, USA) with excitation at 346 nm and emission at 442 nm. When the donor is excited, the energy is transferred to the quencher and no fluorescence is emitted. If this peptide is cleaved by NEP or IDE, the quencher can no longer quench the fluorescence, so strong fluorescence emission is detected.

### Aβ-degrading activity assay using qf-Aβ(12-16)AAC as substrate

qf-Aβ(12-16)AAC (sequence VHHQKAAC with Alexa-350 and Dabcyl attached to Cys sidechain and the N-terminus, respectively) was synthesized according to the previously published procedures^28^ and dissolved as a 2 mM stock solution in DMSO. SH-SY5Y cells were seeded in 24-well plates (450 μL; cell density 1 × 10^6^ cells/mL) in DMEM plus 10% FBS and treated with the compounds as above for 12 h, then the medium was replaced with assay buffer containing 4 μM qf-Aβ(12-16)AAC and the cells incubated for 1 h. To measure Aβ-degrading activity, 150 μL of the buffer was assayed for fluorescence as above.

### Protease inhibition assay

SH-SY5Y cells seeded in 12-well plates (1 mL; cell density 1 × 10^6^ cells/mL) were incubated with vehicle or compound 8 for 12 h, then the medium was replaced with 400 μL of assay buffer containing 50 μM thiorphan (Sigma) and the cells incubated for 30 min, then 100 μL of the buffer was removed and mixed with 0.8 μL of 2 mM qf-Aβ(12-16)AAC, and the mixture returned to the well. The cells were then incubated for 1 h and 150 μL of the supernatant taken for fluorescence measurement as above.

### NEP western blotting

SH-SY5Y cells were seeded on 100-mm culture dish at a cell density of 5.5 × 10^6^ cells/mL (about 60–70% confluent) in DMEM/F12 medium with 1% FBS and the medium volume of 10 mL. Retinoic acid (RA) was added the day after plating at a final concentration of 10 μM in DMEM/F12 medium with 1% FBS. The medium was replaced with fresh DMEM/F12 medium containing 10 μM RA every two days. After 5 days in the presence of RA, cells were ready for NEP expression measurements. The culture medium was replaced with fresh DMEM/F12 medium supplemented with 1% FBS and 5 μM of the indicated compounds or vehicle, and the cells were incubated for 24 h. Cells were then washed out of the dishes with PBS and centrifuged at 200 g for 5 min at 4 °C, and the cell pellets were collected for membrane extraction. The membrane fractions were extracted from the cell pellets using the Mem-PER Plus Membrane Protein Extraction Kit (Thermo Scientific, USA). After quantifying the protein content of the membrane fractions using the BCA kit (Thermo Scientific, USA), 10 μg of protein was resolved on an 8% Bis-Tris gel by SDS-PAGE and transferred to a nitrocellulose membrane (PerkinElmer, USA). The membrane was then blocked in 5% nonfat dry milk (Fonterra, New Zealand), 0.1% Tween 20, 50 mM Tris, 15 mM NaCl, pH 7.5 for 1 h at 4 °C, and incubated overnight at 4 °C with mouse monoclonal anti-human NEP antibody (MAB11821, R&D Systems, USA) and mouse monoclonal anti-glyceraldehyde 3-phosphate dehydrogenase (GAPDH) antibody (Proteintech, USA) at a dilution of 1:5000 in the blocking buffer. After three washes with 0.1% Tween 20, 50 mM Tris, and 15 mM NaCl (pH 7.5), the membrane was incubated for 2 h at 4 °C with horseradish peroxidase-conjugated anti-mouse IgG antibodies (R&D Systems, USA) at a dilution of 1:1000 in the blocking buffer.

To compare the mouse brain NEP protein levels, the cortex pellet from formic acid extraction was collected and washed twice with PBS. The membrane fractions of the tissue pellets were extracted by stirring with 400 μL of the buffer (50 mM Tris, 150 mM NaCl, 5 mM EDTA, 0.5% Triton X-100, 0.5% sodium deoxycholate, pH 8) for 40 min at 4 °C. After stirring, the solution was centrifuged at 20000 g for 15 min at 4 °C. The supernatant containing extracted membrane proteins was collected and concentrated by Amicon Ultra-0.5 (cutoff 30 kDa, Millipore, USA). After quantifying the protein content using the BCA kit (Thermo Scientific, USA), 12 μg of protein was analyzed by SDS-PAGE and immunoblotted as described above, substituting rat monoclonal anti-mouse NEP antibody (MAB1126, R&D Systems, USA) (1:500 dilution) as primary antibody and horseradish peroxidase-conjugated anti-rat IgG antibodies (R&D Systems, USA) (1:1000 dilution) as secondary antibody.

### Animal experiments

All animal experiments were approved by the Institutional Animal Care and Use Committee of the Academia Sinica. The mice were kept in individually ventilated cages (IVC) on a 12:12 h light:dark cycle with food and tap water freely available.

To analyze brain Aβ content, APP_swe_/PS_1_dE_9_ transgenic mice (B6C3-Tg(APP_swe_, PSEN_1_dE_9_)85Dbo/Mmjax) were purchased from Jackson Laboratories (USA) and bred and genotyped as described on the Jackson website^44^,^45^. Five APP_swe_/PS_1_dE_9_ mice were fed orally with vehicle and five with compound 7 (10 mg/kg/day) six times a week by gavage from the age of 4.5–5.5 months for 6.5 months. In the first 3 months, the compound was dissolved at a concentration of 1 mg/mL in 2% carboxymethylcellulose, after which the solvent was changed to the mixture of PEG 600 and Cremophor EL (v/v ratio 2:1) containing 1% methylcellulose for better solubility. The control five APP_swe_/PS_1_dE_9_ mice were fed the corresponding solvent.

In the short-term treatment test, compound 1, 7, or 8 was dissolved at a concentration of 1 mg/mL in the mixture of PEG 600 and Cremophor EL (v/v ratio 2:1) containing 1% methylcellulose, then the compound or solvent was fed to 5-month-old B6C3 mice (non-transgenic female littermates of the APP_swe_/PS_1_dE_9_/B6C3 matings) (10 mg/kg/day, five mice per group) by gavage daily for 7 days. The mice were sacrificed 1 h after the last feed. The brains were removed, and the cortical and hippocampal tissues were dissected out and placed in Eppendorf tubes, immediately frozen in liquid nitrogen, and stored at −80 °C until use.

To analyze Aβ plaque in the brain, APP_swe_/PS_1_dE_9_ transgenic mice were fed orally with vehicle (n = 3, male mice) or compound 8 (n = 6, male mice) six times a week (10 mg/kg/day) by gavage from the age of 3 months to 11 months. Then, the mice were euthanized by cervical dislocation. These mice were perfused transcardially with PBS (136.89 mM NaCl, 2.68 mM KCl, 1.62 mM KH_2_PO_4_, and 10.14 mM Na_2_HPO_4_, pH 7.4) buffer, and post-fixed in 4% paraformaldehyde (PFA)/PBS solution with gentle shaking at room temperature for 3 days. After post-fixation, brain samples were washed with PBS buffer and preserved in 70% ethanol/water solution.

### Protein extraction for Aβ ELISA

Half of the frozen cortical and hippocampal tissue samples were homogenized in 1 mL tapered tissue grinders in 400 μL of ice-cold TBS buffer (50 mM Tris, 150 mM NaCl, 2 mM EDTA, pH 7.4) containing a protease inhibitor cocktail (1:100 dilution) (P8340, Sigma), then the homogenates were transferred to 1.5 mL Eppendorf tubes and centrifuged at 20000 g for 20 min at 4 °C. The supernatant contained soluble Aβ, whereas the TBS-insoluble pellet contained insoluble Aβ. The TBS-insoluble pellet was suspended in 70% formic acid, sonicated for 1 min, then centrifuged at 20000 g for 20 min at 4 °C. The final supernatant containing the solubilized Aβ was removed and neutralized with 20 volumes of 1 M Tris base. The protein concentration of samples containing the solubilized “insoluble” Aβ was quantified using the Bradford protein assay (Bio-Rad #500-0006).

### Aβ40 and Aβ42 ELISA

The samples containing the solubilized “insoluble” Aβ were diluted 800 fold before ELISA quantification, then Aβ40 and Aβ42 were quantified using human Aβ40 and Aβ42 ELISA kits (Invitrogen, Catalog no. KHB3481 and KHB3441) according to the manufacturer’s protocol, measuring the absorbance at 450 nm on a SpectraMax^®^ Paradigm^®^ Multi-Mode reader (Beckman/Molecular Devices, USA). All samples were analyzed in triplicate.

### Real-time PCR Analysis

Half of the frozen cortical and hippocampal tissues from APP_swe_/PS_1_dE_9_ transgenic mice (long-term treatment, n = 5 for each group) and half of the whole brain from B6C3 mice (short-term treatment, n = 5 for each group) was immersed in TRIzol^®^ reagent (Invitrogen) for 1 h, then homogenized as above using a tapered tissue grinder. Total RNA was purified using a TRIzol^®^ Plus Reagent RNA Purification Kit (Invitrogen) according to the manufacturer’s protocol. All RNA samples were then treated with DNase I to remove any residual DNA. Reverse transcription of 5 μg of RNA was carried out using the Maxima First Strand cDNA Synthesis Kit (Thermo Scientific, USA), with β-actin as the reference gene. The sequences of the primers used were: (a) NEP, forward, TCCTGACTATCATAGCGGTGAC; reverse, GACGTTGCGTTTCAACCAGC, (b) β-actin, forward, GGCTGTATTCCCCTCCATCG; reverse, CCAGTTGGTAACAATGCCATGT. Quantitative PCR was performed on 2 μg of cDNA in a total volume of 20 μL in a Light Cycler 480 II (Roche) using a Multicolour PCR detection system and SYBR Green. The thermal cycler conditions were activation at 95 °C for 5 min, followed by 45 cycles of amplification containing three steps: (1) denaturation at 95 °C for 10 sec; (2) annealing at 60 °C for 10 sec, and (3) extension at 72 °C for 10 sec. NEP mRNA levels in each sample were normalized to β-actin mRNA levels. All samples were analyzed in triplicate.

### Thioflavin S (ThS) staining

PFA-fixed left hemispheres were dehydrated by a semi-enclosed benchtop tissue processor (Leica TP1020, Germany). Paraffin blocks containing the dehydrated samples were prepared by paraffin embedding module (Leica EG1150H). Coronal sections with 3 μm thickness were cut on a rotary microtome (Leica RM2235, Germany). Paraffin slides were deparaffinized and rehydrated with xylene, absolute ethanol, 95% ethanol, 70% ethanol, and water, sequentially. Rehydrated slides were applied with 1% (w/v) thioflavin S solution for 10 min at room temperature (protected from light). The slides were washed with 80% ethanol and water to remove excess fluorochrome and to facilitate visualization. Thioflavin S-positive signals were then visualized by a fluorescence microscope. The plaque number and plaque area were analyzed by ImageJ^46^.

### DNA methylation analysis by bisulfite genomic sequencing

Genomic DNAs from the N2a cells with and without the treatment of compounds 1, 7, and 8 were prepared using a QIAamp DNA Mini Kit (Qiagen GmbH, Hilden, Germany). The bisulfite modification method was employed to determine the methylation status of cytosine residues in the genomic DNA^47^. Bisulfite modification of the genomic DNA samples was achieved using an EpiTect Bisulfite Kit (Qiagen, Germany). Nested PCR was used to amplify the upper strand of the bisulfite-modified DNA segment that encompasses the promoter the *NEP* gene. The first round of PCR was performed using the forward primer, 5′-AAGTTTTTTTGGGAAGGGTAAGGTGGG-3′, and the reverse primer, 5′-CCRCCCRCTCTATCCTCCTATTACC-3′, for the modified sequences. The second round of PCR was performed using the forward primer, 5′-AATTTTTAGGTTATTTAGGGAATTGT-3′, and the reverse primer, 5′-AAACRACTAAACAAACACATCCC-3′ for the modified sequences^37^, which amplify the promoter region of the *NEP* gene. The PCR-amplified DNA segments were cloned into the pGEM-T Easy vector (Promega Corp., Madison, USA). Ten clones from the PCR-amplified product were selected for each sample and their sequences determined in order to estimate the incidence of methylation at the CG dinucleotides in the promoter region of the *NEP* gene. In total, 39 CpG sites were analyzed.

### Ethics statement

The animal experiments were approved by the Institutional Animal Care and Use Committee of Academia Sinica. The methods were carried out in accordance with the approved guidelines.

## Additional Information

**How to cite this article**: Chen, P.-T. *et al*. Polyhydroxycurcuminoids but not curcumin upregulate neprilysin and can be applied to the prevention of Alzheimer’s disease. *Sci. Rep.* **6**, 29760; doi: 10.1038/srep29760 (2016).

## Supplementary Material

Supplementary Information
