# Supplementary material for: Polyhydroxycurcuminoids but not curcumin upregulate neprilysin and can be applied to the prevention of Alzheimer’s disease
Source: Sci Rep. 2016 Jul 13;6:29760. doi: 10.1038/srep29760 (PMC4942833; doi:10.1038/srep29760)
Supplement: Supplementary Information [file srep29760-s1.pdf]

Polyhydroxycurcuminoids but not curcumin upregulate neprilysin and can be applied to the prevention of Alzheimer’s disease

Po-Ting Chen[†‡], Zih-ten Chen[‡], Wen-Chi Hou[§], Lung-Chih Yu[†], and Rita P.-Y. Chen[†‡]*

[†] Institute of Biochemical Sciences, National Taiwan University, No. 1, Sec. 4, Roosevelt Rd., Taipei 106, Taiwan

[‡] Institute of Biological Chemistry, Academia Sinica, No. 128, Sec. 2, Academia Rd., Nankang, Taipei 115, Taiwan

[§] Graduate Institute of Pharmacognosy, Taipei Medical University, No. 250, Wuxing St.,Taipei 110, Taiwan

Supplementary Information

Table S1. Statistic analysis of DNA methylation in high CpG density region of the NEP promoter (site 23-34) by Fisher's exact test.

	Unmethylated CpG	Methylated CpG	Ratio of CpG methylation	*p*-value (vs Control)	*p*-value (vs Curcumin)
**Control**	9	111	92.50 %	-	1.0000
**Curcumin**	10	110	91.67 %	1.0000	-
**Compound No. 7**	19	101	84.17 %	0.0688	0.1119
**Compound No. 8**	13	107	89.17 %	0.5030	0.6618
